# Synthesis and Mesomorphic and Electrical Investigations of New Furan Liquid Crystal Derivatives

**DOI:** 10.3389/fchem.2021.711862

**Published:** 2021-09-16

**Authors:** Laila A. Al-Mutabagani, Latifah A. Alshabanah, Sobhi M. Gomha, Tariq Z. Abolibda, Mohamed Shaban, Hoda A. Ahmed

**Affiliations:** ^1^ Department of Chemistry, College of Science, Princess Nourah bint Abdulrahman University, Riyadh, Saudi Arabia; ^2^ Department of Chemistry, Faculty of Science, Cairo University, Cairo, Egypt; ^3^ Chemistry Department, Faculty of Science, Islamic University of Madinah, Al-Madinah, Al-Munawwarah, Saudi Arabia; ^4^ Nanophotonics and Applications Labs, Department of Physics, Faculty of Science, Beni-Suef University, Beni-Suef, Egypt; ^5^ Department of Physics, Faculty of Science, Islamic University in Almadinah Almonawara, Medina, Saudi Arabia; ^6^ Chemistry Department, College of Sciences, Yanbu, Taibah University, Yanbu, Saudi Arabia

**Keywords:** Schiff base/ester, furan liquid crystals, mesophase stability, optical properties, electrical properties

## Abstract

New homologues set liquid crystalline materials, based on furfural derivatives, namely, (E)-4-((furan-2-ylmethylene)amino)phenyl 4-alkoxybenzoate (**F** *n*), were synthesized and investigated for their mesomorphic and optical characteristics. The prepared homologues series constitutes three derivatives that bear different terminal flexible alkyl chain lengths that vary between 6 and 12 carbons and attached to the phenyl ring linked to the ester group. A furfural moiety is introduced into the other terminal of the molecular structure. Mesomorphic characterizations of the prepared derivatives were measured using differential scanning calorimetry (DSC) and polarized optical microscopy (POM). Molecular structures were elucidated via elemental analyses, FTIR, and NMR spectroscopy. DSC and POM showed that all the synthesized furfural derivatives are purely nematogenic, exhibiting an enantiotropic nematic (N) mesophase, except for the longest chain derivative (**F** *12*) that is dimorphic possessing a monotropic smectic A phase and an enantiotropic N mesophase. Results indicated that the incorporation of the heterocyclic furfural ring into the molecular skeleton affected both the mesophase range and stability of investigated homologue. Analysis of the optical properties revealed that the shortest chain compound (**F** *6*) possesses two direct band gaps, at 2.73 and 3.64 eV, in addition to higher absorption than the higher homologues, **F** *10* and **F** *12*. On the other hand, all the synthesized homologues (**F** *n*) showed Ohmic behaviors, with electric resistances in the GΩ range. The values of the electrical resistances are 103.71, 12.91, and 196.85 GΩ at 0.05 V for **F** *6*, **F** *10*, and **F** *12*, respectively.

## Introduction

Today, the higher cost and the lower conversion efficiency of solar energy limit its application. One of the most essential ways to reduce the cost and increase the conversion efficiency is the investigation of various basic organic derivatives to be used in solar devices (Zhang et al., 2018; Li et al., 2019). For solar energy applications such as catalytic photodegradation of dyes, solar hydrogen generation, photo-electrochemical water splitting, and solar cells, bandgap engineering and optical property control are critical parameters (Ahmed et al., 2020a; Helmy et al., 2020; Mohamed et al., 2020; Shaban and El Sayed, 2020; Shaban et al., 2020). Liquid crystalline (LC) semiconductors have been extensively investigated due to their pi-conjugated properties; thus, large numbers of LC materials have been designed for solar cell applications (Frédéric et al., 2012; Funahashi, 2014; Paul, 2016; Hu et al., 2020).

Among the interests to develop new mesogenic cores, the introduction of the fused heterocycle moiety to the molecular structure has been extensively investigated, and new various mesomorphic properties have been resulted (Han, 2013; Ghosh and Lehmann, 2017). The addition of heteroatoms such as nitrogen, sulfur, or oxygen not only increases the species of the liquid crystals but also greatly impacts the thermal and geometrical parameters of investigated materials (Frédéric et al., 2012; Han, 2013; Paul, 2016; Ghosh and Lehmann, 2017; Merlo et al., 2018; Yeap et al., 2018; Weng et al., 2019a; Weng et al., 2019b; Ren et al., 2019). Fused aromatic ring insertion into central or terminal structure leads to a change in polarizability, dipole moment, geometry, and consequently mesophase transition temperatures as well as the dielectric constant (Seed, 2007; Ha et al., 2010; Tariq et al., 2013; Merlo et al., 2018; Yeap et al., 2018; Weng et al., 2019a; Weng et al., 2019b; Ren et al., 2019; Nafee et al., 2020). Attachment of electronegative heteroatoms (S, N, and O) can significantly influence the bond angle as well as the delocalized resonance and the molecular geometry (Titov and Pavlyuchenko, 1980; Lehmann et al., 2005; Takase et al., 2007; Gomha and Riyadh, 2011; Gomha et al., 2016a; Gomha et al., 2016b; Gomha et al., 2020; Nafee et al., 2020; Sayed et al., 2020; Abu-Melha et al., 2021; Gomha et al., 2021a).

Mesomorphic properties of an organic material are principally determined by its molecular shape. Minor changes in the molecular structure result in significant changes in mesomorphic behavior (Alnoman et al., 2020; Zaki et al., 2020; Khushaim et al., 2021). The Schiff base (CH = N) serves as a linking bridge between the rigid core moieties. Despite having a stepped core structure, it also maintains molecular linearity, resulting in greater thermal stability and enabling the formation of phase (Ahmed et al., 2020b; Hagar et al., 2020). Recently, many mesomorphic Schiff base/ester systems of low molecular mass have been designed and examined (Jakeman and Raynes, 1972; Cîrcu et al., 2011; Al-Mutabagani et al., 2021a; Altowyan et al., 2021; Al-Mutabagani et al., 2021b; Gomha et al., 2021b; El-Atawy et al., 2021). The connection of -COO– and –CH = N– linking groups and terminal long flexible chain provides flexibility to the molecule, which leads to conformational changes during the phase transition. Furthermore, Schiff base/ester-based derivatives have many photo-switchable technological applications (Lehmann et al., 2005; Takase et al., 2007).

**SCHEME 1:**
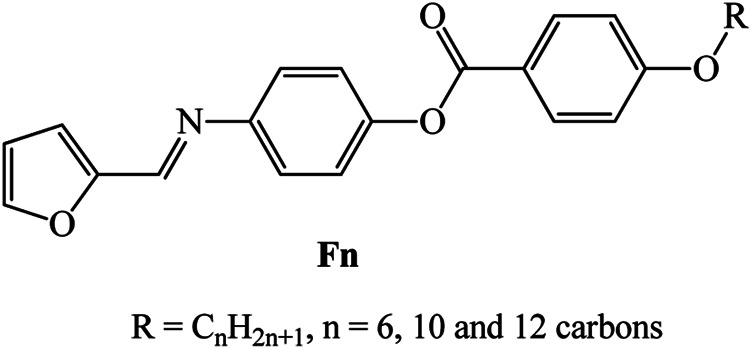
Molecular structure of investigated series, **F** *n*.

In a previous work (Petrov and Pavluchenko, 2003), the physicochemical properties of (E)-4-((furan-2-ylmethylene)amino)phenyl 4-heptyloxybenzoate were investigated, and the results indicated it possesses nematic phase monotropically with lower thermal stability. Herein, the goal of the present work is to first develop homologues series of azomethine derivatives bearing terminal heterocyclic furan moiety, with different terminal alkoxy chain length, for example, (E)-4-((furan-2-ylmethylene)amino)phenyl 4-alkoxybenzoate, **F** *n* (Scheme 1). On the furan ring, a -CH = N- linkage is present, while the other terminal has an ester linkage with a phenyl ring attached to alkoxy groups of different length.

Recently, in our research laboratory, we focused our attention on the solar energy investigations of new synthesized optical materials to correlate their mesomorphic behavior with the energy measurements. So, the second aim of the investigation is to investigate the mesomorphic and optical properties of the present system and study the effect of the change terminal length of flexible chains on their mesomorphic behavior. Furthermore, the study also aims to investigate the electric and optical properties, the electric resistance, conductance, energy gap, as well as Urbach energy.

## Experimental

### Synthesis

**SCHEME 2:**
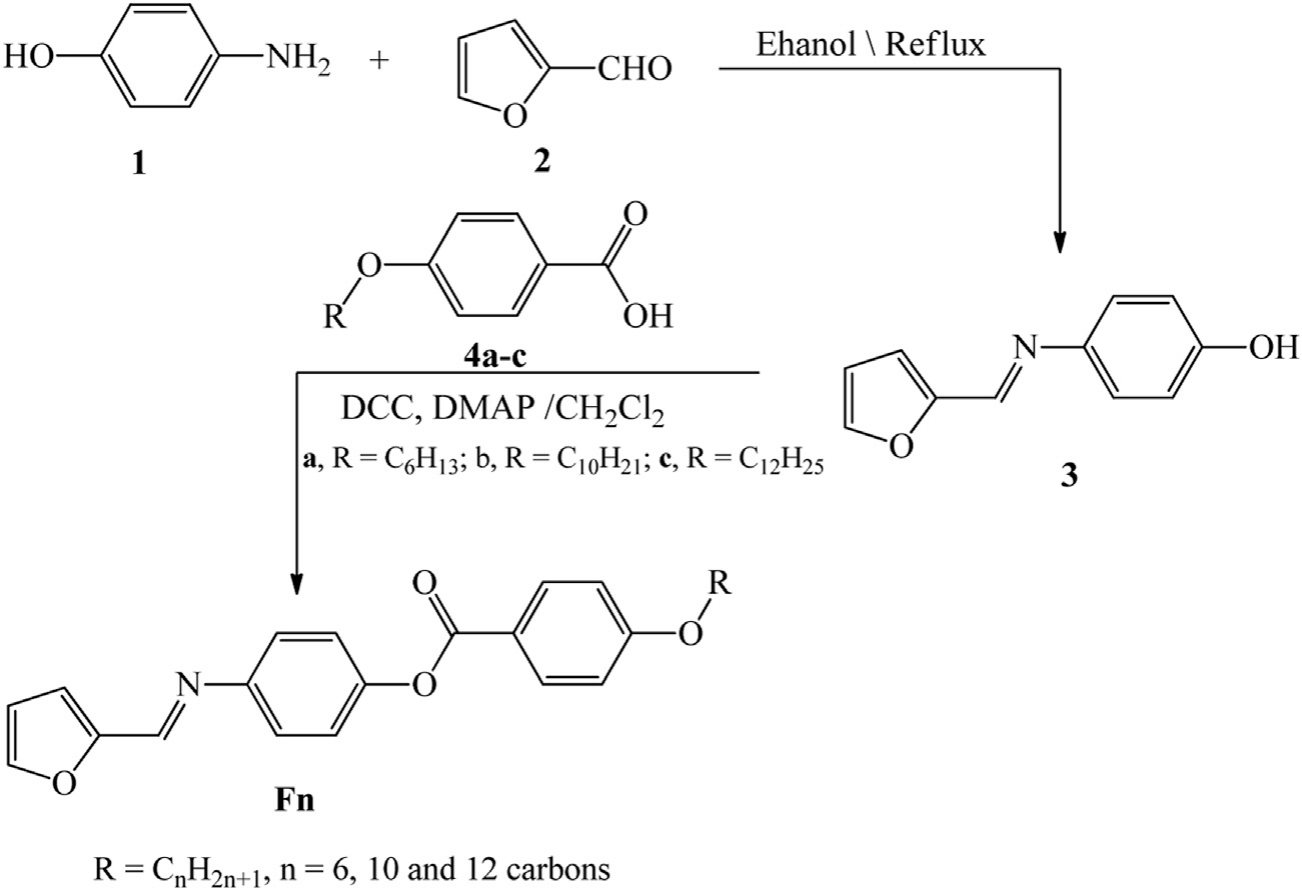
Synthesis route of title compounds **F** *n*.

The present homologue **F** *n* was designed as shown in the following Scheme 2:

The synthetic procedures, and physical and chemical characterizations of products **F** *n* are listed in supplementary data.

## Results and Discussion

### Liquid Crystalline Investigations

**FIGURE 1:**
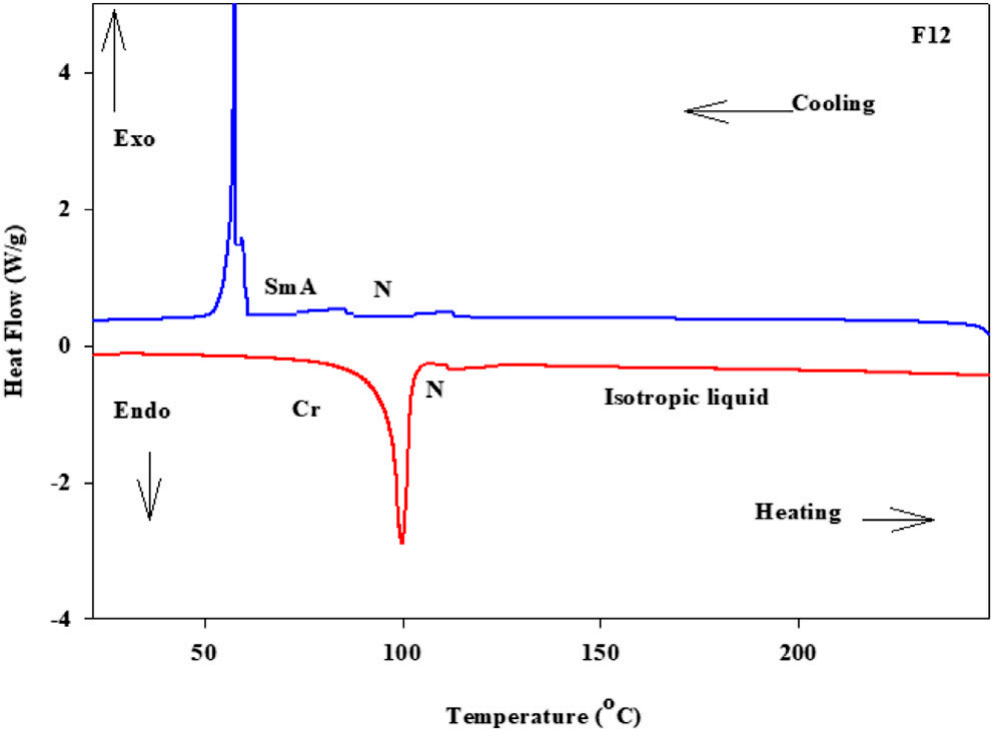
DSC thermograms of **F** *12* which are measured from the 2^nd^ heating/cooling cycles at a rate of ±10°C/min.

**FIGURE 2:**
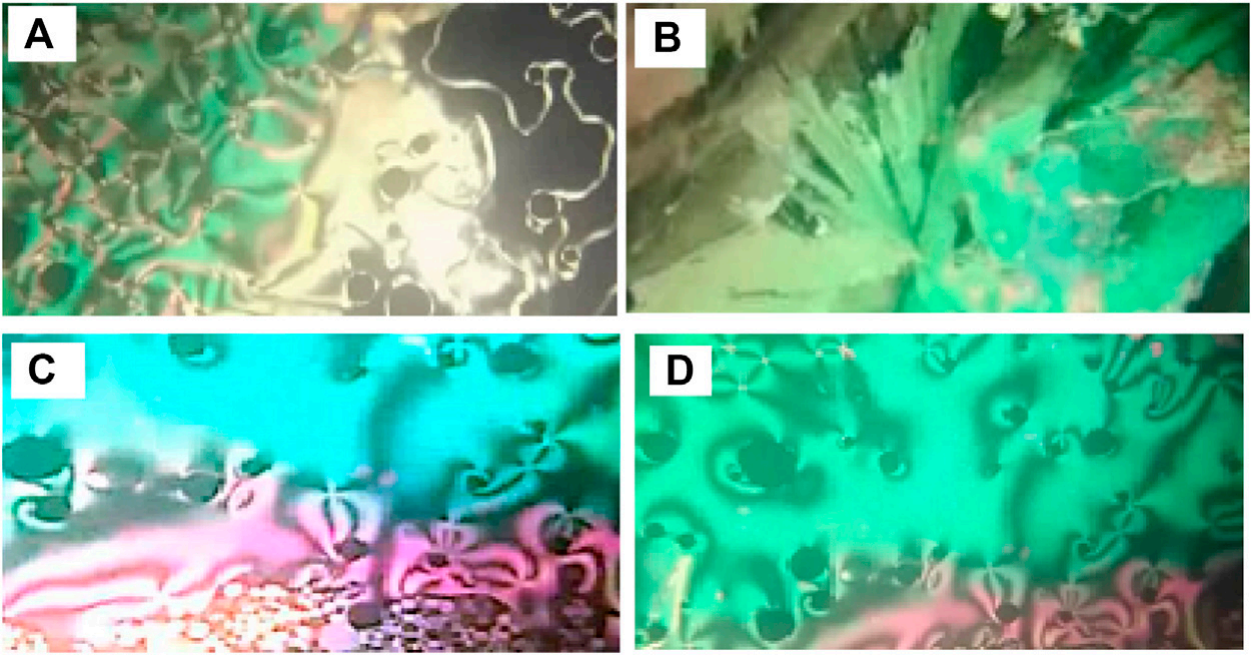
Images detected by POM for **(A)** derivative **F** *12* of N phase at 105.0°C on heating; **(B)** derivative **F** *12* of SmA phase at 84.0°C on cooling; **(C)** derivative **F** *6* of N phase at 135.0°C on heating; **(D)** derivative **F** *10* of N phase at 120.0°C on heating.

**FIGURE 3:**
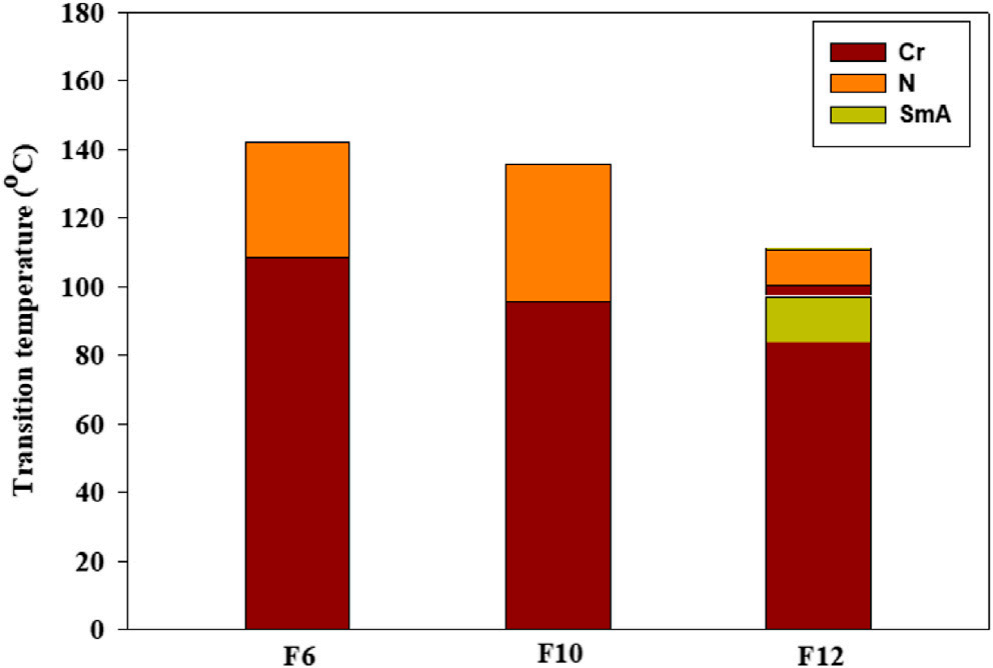
Impact of terminal alkoxy chain length (*n*) on the mesomorphic transitions of the investigated furan derivatives, **F** *n*.

Phase transitions and optical characteristics of the investigated furfural group have been investigated by DSC and POM measurements. A DSC thermogram of designed compound **F** *12* is illustrated in Figure 1 *via* heating/cooling cycles, as a representative example. It can be observed from Figure 1 that two transition peaks observed vary according to the geometrical shape of designed materials, **F** *n*. Moreover, the mesomorphic transitions from Cr→ N upon heating and I→ N upon cooling were observed as well. Significant endothermic and exothermic peaks depending on the length of the attached terminal flexible-chain were also observed. These peaks are ascribed to mesomorphic transitions and the cooling scan affirmed those observations upon decrement in the temperature. Optical textures under POM confirm the DSC results. Figure 2 illustrates the POM images of the **F** *12* derivative. The mesomorphic transition temperatures, as driven from measurements of DSC, and their enthalpies of transition for all the designed furfural members, **F** *n*, are summarized in Table 1. In order to investigate the impact of the length of the terminal alkoxy chain (n) on the mesomorphic behavior of formed compounds, Figure 3 depicts their relations. Table 1 and Figure 3 reveal that all the synthesized members of heterocyclic derivatives **F** *n* are mesomorphic in nature with high mesomorphic thermal stability and good mesophase range dependent on their terminal flexible chain length. Moreover, compounds **F** *6* and **F** *10* are enantiotropic possessing pure N phase, while the longer chain derivative **F** *12* possesses two mesomorphic transitions (dimorphic). The dimorphic property of **F12** indicates that it possesses both the monotropic (less stable phase SmA) and enantiotropic N mesophase. It can also be seen from Table 1 and Figure 3 that the melting temperature of compounds varies randomly with the chain length (*n*). The shortest terminal length member (**F** *6*) exhibits N phase enantiotropically, with a nematic thermal stability and temperature of 141.8 and 33.3°C, respectively. For the **F** *10* derivative, it also possesses the enantiotropic N mesophase, with a nematogenic stability and the highest temperature of nearly 135.6 and 39,9°C, respectively, while the longest chain length derivative (**F** *12*) possesses less thermal N stability (110.4°C) and induced smectic A mesophase. Moreover, its smectogenic stability is nearly 86.0°C and its total mesomorphic stability (SmA and N phases) nearly 10.3°C. In general, the architecture of the molecule, polarizability, and the dipole moment of the designed materials are highly impacted by the electronic nature of the terminal substituents. In addition, the mesomorphic behavior is enhanced by the increase in polarity and/or polarizability of the molecular mesogenic parts. The mesomorphic range of present investigated homologues increases in the order as follows: **F** *12* > **F** *6* > **F** *10*. The mesomorphic behavior of the present rod-like molecules directly affects the molecular–molecular interactions that depend upon the geometrical shape of the polar terminal groups and the heterocyclic moieties in the molecule (Nafee et al., 2020). Mesomorphic property observations indicate the sharing of these factors in different extents. The end-to-end aggregation attributed to the oxygen of alkoxy chain and the carbonyl ester moiety and the side-by-side cohesive forces between molecules are important factors that determine the type of the observed mesophase (Gray, 1962; Luckhurst and Gray, 1979).

**TABLE 1:** Transition temperatures of mesophases, °C (transition enthalpy **ΔH**, kJ/mole), mesomorphic range (Δ**T**, °C), and the normalized entropy of transition, **ΔS**/R, for present series **F** *n.*

Compound	*T*_Cr-SmA_	*T*_Cr-N_	*T*_SmA-N_	*T*_N-I_	*ΔT*	*ΔS*_N-I_/R
**F** *6*	–	108.5 (36.80)	–	141.8 (1.48)	33.3	0.43
**F** *10*	–	95.7 (37.79)	–	135.6 (1.72)	39.9	0.51
**F** *12*	100.1 (35.27)	–	86.0 (1.78)*	110.4 (1.43)	10.3	0.45

Cr-N = solid-to-nematic phase transition.

Cr-SmA = solid-to-smectic A phase transition.

SmA-N = smectic A-to-nematic phase transition.

N-I = nematic-to-isotropic liquid phase transition.

*Monotropic phase.

The normalized transition entropy changes, ΔS_N-I_/R, of the present investigated homologues (**F** *n*) are tabulated in Table 1. Results showed very small entropy changes that mainly depend on the kind of terminal substituents and the mesogenic cores. The observed small values can be attributed to their lower anisotropy resulted from their molecular geometry and molecular biaxiality (Attard et al., 1994; Henderson et al., 2005; Yeap et al., 2011; Yeap et al., 2015). Induction, conjugation forces, particular dipolar, and π-π stacking interactions (Attard et al., 1994; Henderson et al., 2005; Yeap et al., 2011; Yeap et al., 2015) all play essential roles in molecular orientation and hence in molecule arrangement and mesophase formation.

X-ray diffraction (XRD) is another characterized tool to confirm the mesophases (Zhang et al., 2004). XRD measurements were performed for compound **F** *12* upon cooling of the sample from 100°C to confirm the presence of the SmA phase (Supplementary Figure S3, Supplementary Data). The XRD pattern at recorded temperature upon cooling of the sample showed only one peak at angle 2Ɵ = 23.0°, assigned to the presence of SmA phase transition. Thus, the XRD and POM results indicated the presence of SmA mesophases monotropically.

### Electrical Properties

**FIGURE 4:**
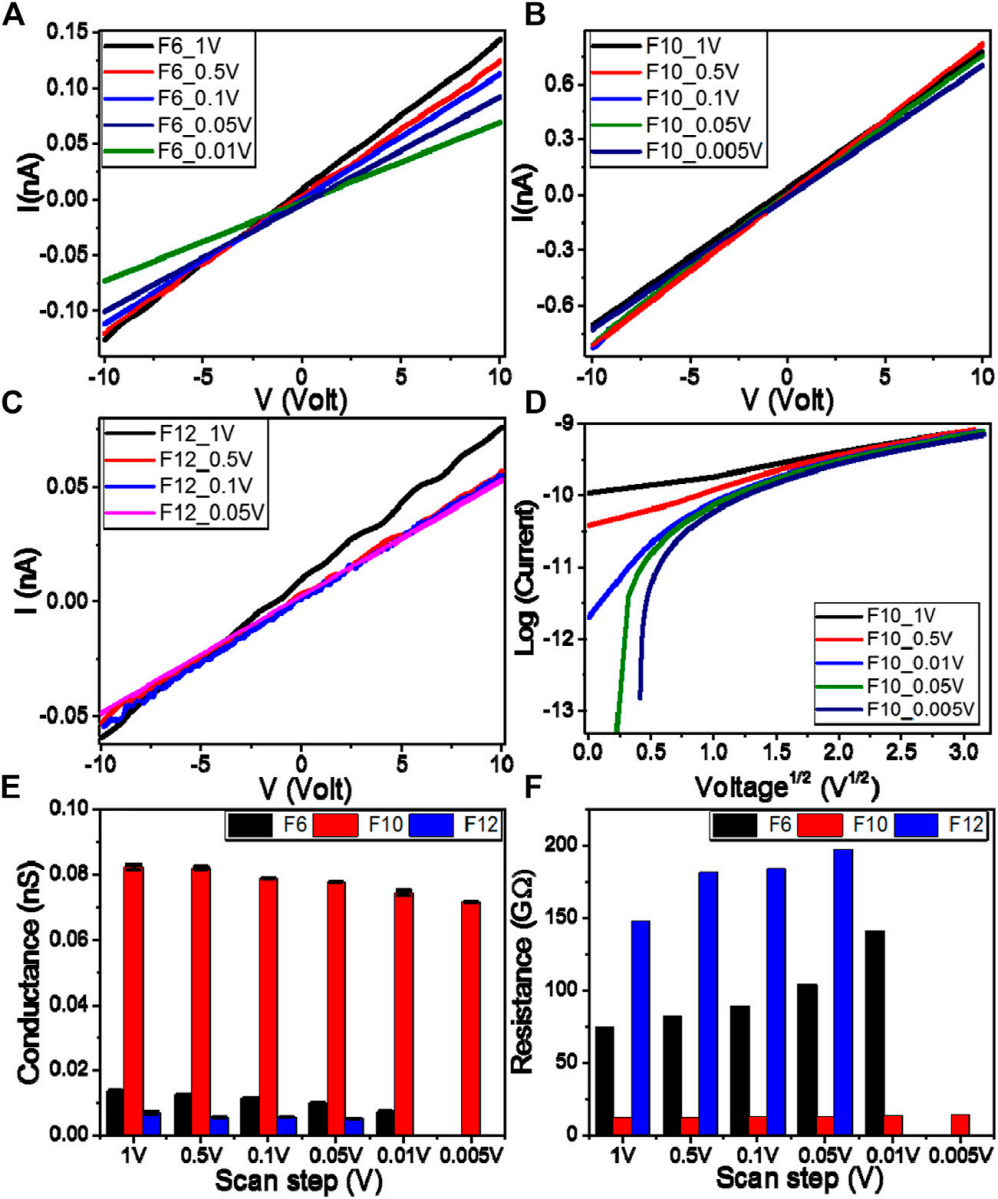
Electrical voltage–current characteristics of **(A) F** *6* **(B) F** *10*, and **(C) F** *12* samples **(D)** Log(I) vs. V^0.5^ for **F** *10* samples **(E)** electric conductance and **(F)** electric resistance at different scan steps for samples **F** *6*, **F** *10*, and **F** *12*.

The Keithley measurement source unit (Model 4200 SMU) is used to test the electrical properties of the investigated films. The samples were provided with Ohmic contacts using silver paste (Resistivity <0.04 Ω.cm). The current–voltage (*I*–*V*) characteristics of the present investigated **F** *6*, **F** *10*, and **F** *12* films are recorded by varying the applied voltage (V) from −10 to 10 V with different scan steps, 1–0.005 V, as shown in Figures (A–C). The behaviors are almost linear (Ohmic behaviors). So, the resistances of the materials are almost constants and independent of the current moving through them. Recent studies have showed that the polymeric and organic systems are of Schottky diode behavior at low voltage. However, in the present investigation, the relation between log(I) and V^1/2^ is nonlinear, as illustrated in Figure 4D, which implies that our films do not follow the Schottky diode behavior. As shown in Figures (C–D), the current depends on the voltage and the scan rate. So, the samples seem to have a capacity of some kind. It could be electronic or perhaps ionic. At a specific voltage, the resistive current only depends on voltage and not on the scan rate. The capacitive current depends on the scan rate. At a given voltage, capacitance can be calculated by dividing the difference in current by the difference in the scan rate, that is, C = (I_1_–I_2_)/([dV_1_/dt]—[dV_2_/dt]) at specific voltage. The obtained mean values of the capacitance are 113.0, 195.3, and 31.5 µF for **F** *6*, **F** *10*, and **F** *12* at *5V*, respectively*.* Figures 4E,F show the obtained values of conductance and resistance, respectively, for all films at different scan steps. Whereas F**6** and **F** *12* have electrical conductance in the same order of magnitude (Figure 4E) **F** *10* films show much higher conductance than either of **F** *6* or **F** *12* films. As shown in Figure 4E, the values of the electrical conductance are 0.0096 ± 0.00005, 0.0774 ± 0.00011, and 0.0051 ± 0.00004 nS at 0.05 V for **F** *6*, **F** *10*, and **F** *12*, respectively. By changing the scan step from 1 to 0.005 V, the electric conductance decreases from 0.0821 ± 0.00095 to 0.0714 ± 0.00006 nS for **F** *10* film. As shown in Figure 4F, the values of the electrical resistances are 103.71, 12.91, and 196.85 GΩ at 0.05 V for **F** *6*, **F** *10*, and **F** *12*, respectively. The resistance of the **F** *10* film is increased from 12.17 to 14.00 GΩ by decreasing the scan step from 1 to 0.005 V. This behavior confirms the formation of wide nematic mesophase according to the molecular interactions within the molecule since electrical conductance depends mainly on the number and mobility of charge carriers (Rathi et al., 2017; Kumar et al., 2018).

### Optical Spectra and Energy Gap Calculation

**FIGURE 5:**
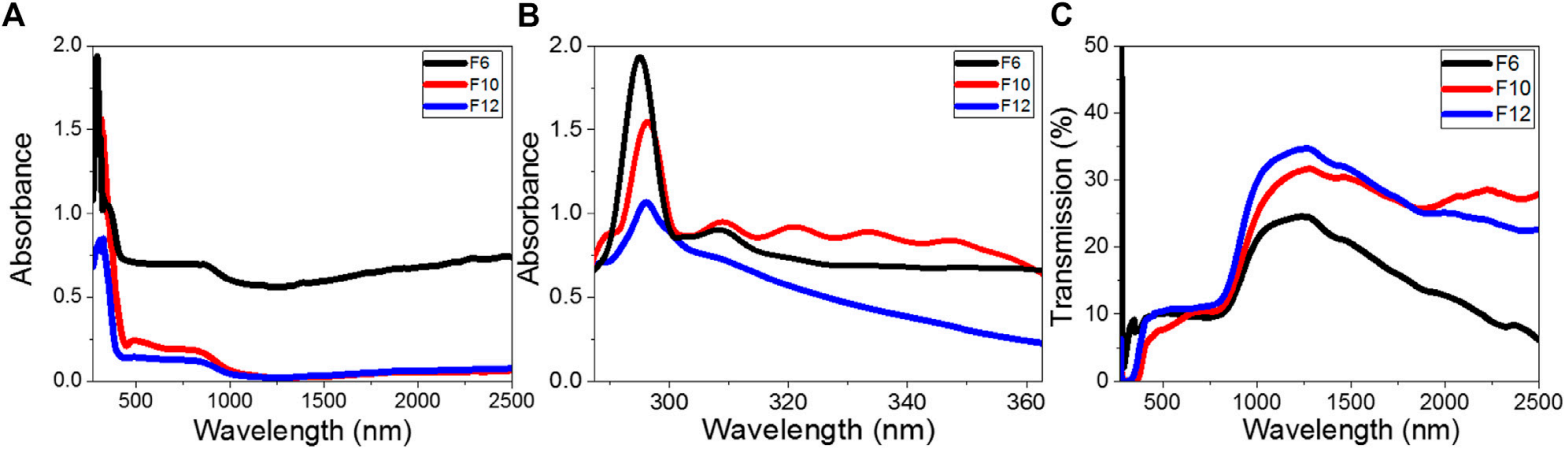
Optical **(A,B)** absorbance and **(C)** transmission spectra of **F** *6*, **F** *10*, and **F** *12* films.

The optical absorbance and transmission spectra of **F** *6*, **F** *10*, and **F** *12* were measured using a PerkinElmer spectrophotometer (Lambda 950 UV-vis-NIR) by a wavelength range from 250 to 2,500 nm utilizing a blank glass substrate in the reference beam. Figures 5(A–C) illustrate the dependence of the transmittance and absorbance spectra of the films on the wavelength. The absorbance spectra in Figure 5A demonstrate a strong absorption behavior for **F** *6* compared to **F** *10* and **F** *12*. All films show strong absorbance up to 420 nm. Then, the absorbance decreases to reach a stable value (plateau) from 430 to 870 nm before decreasing again to reach minimum absorbance at 1,250 nm. As shown in Figure 5B, a strong absorption band is observed at 294 nm for **F** *6* and 296 nm for **F** *10* and **F** *12*. The absorbance intensity increases in the order **F** *6* > **F** *10* > **F** *12*. The left edge of the absorption band is redshifted, leading to a decrease in the full width at half maximum. This redshift is due to the size impacts, where small size decreases the spin–orbit coupling and moderates the exciton locations (Shaban and El Sayed, 2016). The redshift and high absorption values in UV and visible regions are desirable features in energy-efficient solar cells (Liu et al., 2016). Another weak absorption band is observed for **F** *6*, which becomes weaker for **F** *10* and **F** *12*. The small ripples observed for **F** *10* result from faint interference fringes and refer to the homogeneity of the film because the optical behavior mainly depends on its chemical composition and morphology. All films showed transmission less than 10% in the UV/visible range (Figure 5C). Then, the transmission increased exponentially in the near-IR region to reach maxima of 35, 32, and 24% at 1,250 nm for **F** *12*, **F** *10*, and **F** *6*, respectively. After that, the transmission decreased as the wavelength increased.

**FIGURE 6:**
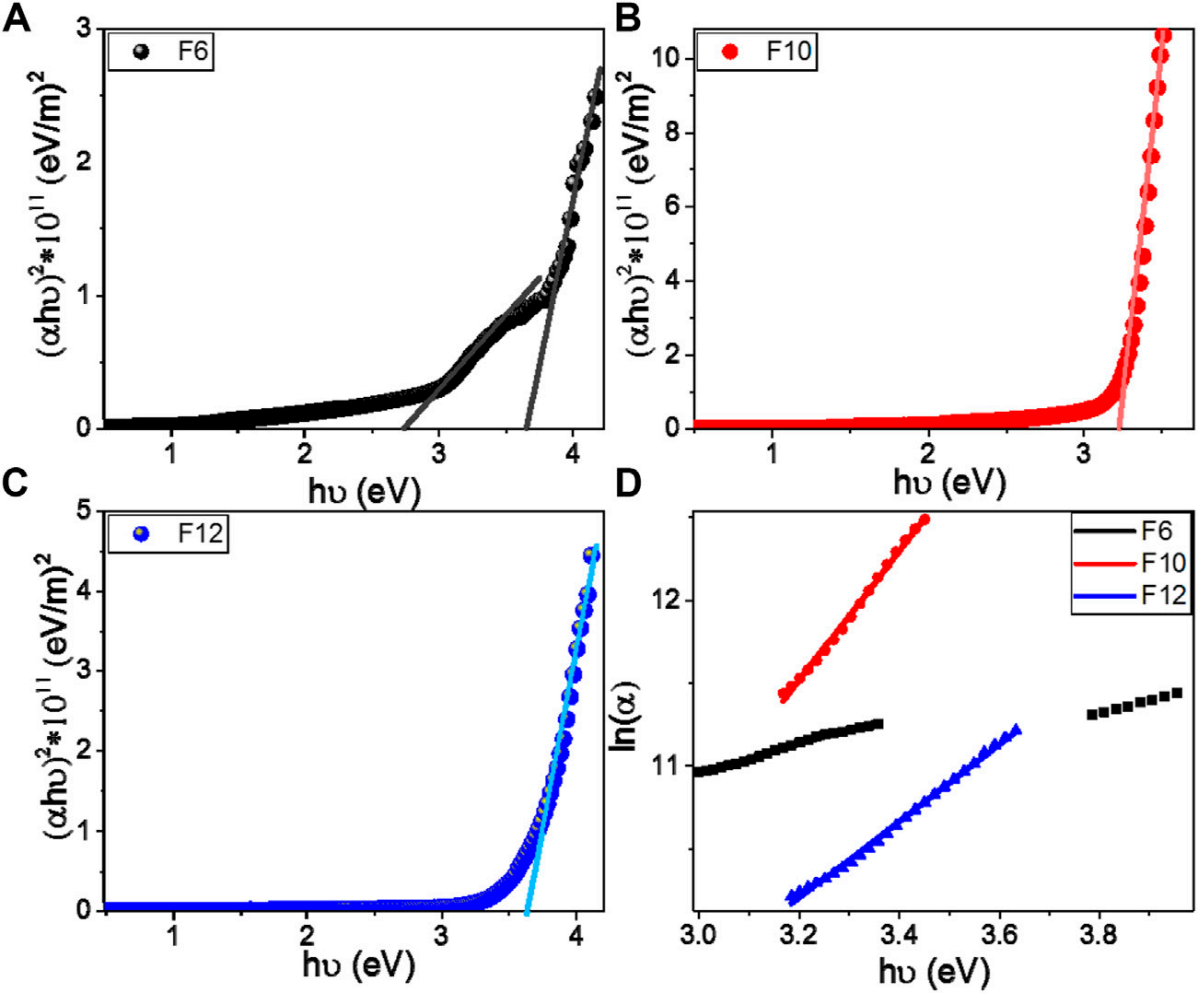
Calculation of energy gap **(A–C)** and Urbach energy **(D)** for **F** *6*, **F** *10*, and **F** *12* films.

According to the optical absorption theorem, the correlation between absorption coefficient, α, and the photon energy, *hν*, for the direct allowed transitions is given by (Shaban and El Sayed, 2015)

$${\left(\alpha \;h\nu \right)}^{2}=A\left(h\nu -Eg\right),$$ (1)

where *h* is Planck’s constant (6.625 × 10^−34^ J/s), *A* is a constant, and *E*_*g*_ is the optical bandgap. The values of direct *E*_*g*_ for **F** *6*, **F** *10*, and **F** *12* are obtained by extrapolating the linear portions of the plot of (*αhν*)^2^ vs. *hν* to *α* = 0, as shown in Figure 6(A–C). The linear parts observed in this figure indicate that the transitions are performed directly. To analyze the region of linearity more carefully, we draw the data only around this photon energy range. Furthermore, to eliminate any personal bias in the estimation and any effect of the vertical shift, we employed the usual linear least square fitting by OriginPro 2018 in the linear region of interest. When the straight line equation is fitted as Y = a + bX, the band gap is equal to the absolute value of the ratio a/b. Interestingly, as reported in Table 2, there are two direct band gaps for the **F** *6* film at 2.73 and 3.64 eV; one direct bandgap for **F** *10* at 3.22 eV and one for **F** *12* at 3.62 eV. The observed reduction in the main bandgap from 3.64 eV for **F** *6* to 3.22 eV for **F** *10* is ascribed to the influence of the density of localized states due to the formation of purely less order nematic phases. This behavior is consistent with the previously reported studies (Mullekom van, 2000). The reduction in the bandgap is very important for solar energy applications, especially photoelectrochemical hydrogen generation and solar cells (Abdelmoneim et al., 2021; Mohamed et al., 2021; Shaban et al., 2021).

**TABLE 2:** Values of the energy gap, Eg, and Urbach energy, *E*_*U*_, of **F** *6*, **F** *10*, and **F** *12*.

Compound	Eg (eV) ± SD	Eu (eV) ± SD	*R* ^2^
**F** *6*	2.73 ± 0.01	1.145 ± 0.017	0.9958
	3.64 ± 0.02	1.323 ± 0.019	0.9981
**F** *10*	3.22 ± 0.02	0.523 ± 0.057	0.9984
**F** *12*	3.62 ± 0.02	0.431 ± 0.025	0.9987

Urbach energy (*E*_*U*_) referred to the width of the exponential absorption edge (the Urbach tail). The tails of the valence and conduction bands are ascribed to the disorder in the material (Mohamed et al., 2021). The exponential dependency of the *E*_*U*_ can be determined according to the following equation (El Sayed and Shaban, 2015):

$$\;\alpha =\alpha_{o}exp\left(\frac{h\upsilon }{E_{U}}\right)\Rightarrow E_{U}={\left[\frac{\delta \left(\ln\left(\alpha \right)\right)}{\delta \left(h\nu \right)}\right]}^{-1},$$ (2)

where *α*_*0*_ is the band tailing parameter that can be obtained by the following equation(Sharma et al., 2014):

$$\alpha_{o}=\sqrt{\frac{\sigma_{0}\left(\frac{4\pi }{c}\right)}{x\;\text{Δ}E}}\;,$$ (3)

where *c* is the speed of light, σ_*o*_ is electrical conductivity at absolute zero, and Δ*E* represents the width of the tail of the localized state in the forbidden gap. Figure 6D shows the plot of ln(α) vs. *h*ν for **F** *6*, **F** *10*, and **F** *12*. The values of *E*_*U*_ were obtained from the slopes of the linear fitting of these curves and are reported in Table 1. The statistical parameters, standard deviation (SD) and correlation coefficient (*R* ^2^), are also reported in this table. The values are 1.145 and 1.323 eV for the two bandgaps of **F** *6* and 0.523 eV for the bandgap of **F** *10*, which refers to the extension of the bandgap edges to cover a wide range of the spectral range.

## Conclusion

Herein, new optical liquid crystalline homologues based on a furfural moiety named (E)-4-((furan-2-ylmethylene)amino)phenyl 4-alkoxybenzoate (**F** *n*) were synthesized. DSC and POM were used to investigate their mesomorphic properties. Mesomorphic and optical examinations revealed that all the designed materials of the furfural set are monomorphic exhibiting enantiotropic N mesophases, except for the longest chain member (**F** *12*) that is dimorphic and possesses monotropic smectic A phase and enantiotropic N mesophase. In addition, the introduction of the heterocyclic moiety into the molecular structure offered the formation of N stability with a good temperature range.

The Keithley measurement source unit and an UV/vis/IR Perkin Elmer spectrophotometer were used for determining the electrical properties of the current investigated series. The shortest chain member (**F** *6*) had shown to possess two direct band gaps of 2.73 and 3.64 eV, in addition to higher absorption than the **F** *10* and **F** *12* derivatives. With electric resistances in the GΩ range, all prepared homologues, **F** *n*, exhibit Ohmic behaviors. Moreover, their electrical resistances are 103.71, 12.91, and 196.85 GΩ at 0.05 V for **F** *6*, **F** *10*, and **F** *12*, respectively. The obtained data revealed that the engineering of the optical band gap and regulation of the electric properties are critical for solar energy applications.

## Data Availability

The original contributions presented in the study are included in the article/Supplementary Material; further inquiries can be directed to the corresponding authors.
