# Supplementary material for: Synthesis and Mesomorphic and Electrical Investigations of New Furan Liquid Crystal Derivatives
Source: Front Chem. 2021 Sep 16;9:711862. doi: 10.3389/fchem.2021.711862 (PMC8503887; doi:10.3389/fchem.2021.711862)
Supplement: Supplementary file 1 [file DataSheet1.pdf]

Supplementary data

Synthesis, Mesomorphic and Electrical Investigations of New Furan Liquid Crystal Derivatives

Laila A. Al-Mutabagani[1], Latifah A. Alshabanah[1], Sobhi M. Gomha[2,3]*, Tariq Z. Abolibda[3], Mohamed Shaban[5,6] and Hoda A. Ahmed[2,6]*

[1]*Department of Chemistry, College of Science, Princess Nourah Bint Abdulrahman University,Riyadh 11671, Saudi Arabia; laalmutbagani@pnu.edu.sa (L.A.A.-M.); laalsabanah@pnu.edu.sa (L.A.A.)*
[2]*Department of Chemistry, Faculty of Science, Cairo University, Cairo 12613, Egypt, ahoda@sci.cu.edu.eg, smgomha@iu.edu.sa*
[3]*Chemistry Department, Faculty of Science, Islamic University in Almadinah Almonawara, Almadinah Almonawara, 42351, Saudi Arabia, t.z.a@iu.edu.sa.*
[4] *Nanophotonics and Applications Labs, Department of Physics, Faculty of Science, Beni-Suef University, Beni-Suef 62514, Egypt, mssfadel@aucegypt.edu*
[5]*Department of Physics, Faculty of Science, Islamic University in Almadinah Almonawara, Almadinah, 42351, Saudi Arabia*
[6]*Chemistry Department, College of Sciences, Yanbu, Taibah University, Yanbu 30799, Saudi Arabia.*

** Correspondence: H. A. Ahmed, ahoda@sci.cu.edu.eg; S.M. Gomha, smgomha@iu.edu.sa*

1. *Materials*

4-Hexyloxybenzoic acid, 4-decyloxybenzoic acid, 4-dodecyloxybenzoic acid 4-4-aminophenol and furfural, were purchased from Sigma Aldrich (Germany). dichloromethane, *N,N'*-dicyclohexylcarbodiimide (DCC), ethanol and 4-dimethylaminopyridine (DMAP) were purchased from Aldrich (Wisconsin, USA).

2. *Synthesis of* (E)-4-((furan-2-ylmethylene)amino)phenol (**3**)

A mixture of furfural (0.96g, 10 mmol) and 4-aminophenol (1.09g, 10 mmol) in ethanol (20 mL) were refluxed for two hours (monitored by TLC). The mixture was cooled to room temperature and filtered. The obtained solid was washed was cold ethanol and recrystallized twice from hot methanol to give pure imine compound **3** as indicated by TLC analysis**.** Mp 199-201 °C (Lit mp 197-199 °C) [i].

3. *Synthesis of (E)-4-((furan-2-ylmethylene)amino)phenyl 4-alkoxybenzoate, **Fn***

A mixture of imine compound **3** (1.87g, 10 mmol) and the appropriate 4-alkoxy benzoic acid derivatives **4** (10 mmol for each) in dry methylene chloride (25 mL) containing *N*, *N′*–dicyclohexylcarbodiimide (DCC, 10 mmol) and few crystals of 4–dimethylaminopyridine (DMAP), as catalyst, were left to stand for 72 hours at room temperature with continuous stirring. The solid separated was then filtered off and the solution evaporated. The solid residue obtained was recrystallized from ethanol to give TLC pure products. The purity of the prepared samples was checked with thin-layer chromatography (TLC) using TLC sheets coated with silica gel (E Merck), and CH_2Cl_2/CH_3OH (9:1) as eluent, whereby only one spot was detected by a UV-lamp. Infrared spectra (IR), Mass, ^{1}H-NMR, and elemental analyses for compounds investigated were consistent with the structures assigned. ^{1}H-NMR data showed the expected integrated aliphatic to aromatic proton ratios in all compounds investigated.

The physical data of products **F***n* are listed below:

(E)-4-((Furan-2-ylmethylene)amino)phenyl 4-(hexyloxy)benzoate (F*6*):

Yield: 84.6%; mp 157-159°C, FTIR (ύ, cm^{-1}): 3083, 2928 (C-H), 1723 (C=O), 1619 (C=N). ^{1}H-NMR (400 MHz, DMSO): *δ*/ppm: 0.80-0.82 (t, 3H, $\underline{CH_3}(CH_2)_3CH_2CH_2O$-), 1.37-1.71 (m, 6H, $CH_3\underline{(CH_2)_3}CH_2CH_2O$-), 1.76-178 (m, 2H, $CH_3(CH_2)_3\underline{CH_2}CH_2O$-), 4.05-4.07 (t, 2H, $CH_3(CH_2)_3CH_2\underline{CH_2}O$-), 6.56-6.59 (t, 1H, furan-H4), 6.68-6.69 (d, 1H, furan-H3), 7.06-7.33(m, 6H, Ar–H), 7.92-7.93 (d, 1H, furan-H5), 8.02-8.05 (d, 2H, Ar–H), 8.44 (s, 1H, CH=N); ^{13}C-NMR (400 MHz, DMSO): *δ*/ppm: 13.97 (CH_3), 22.19, 24.01, 28.97, 34.48, 68.08 (CH_2), 106.69, 114.39, 118.15, 121.06, 122.83, 132.01, 137.01, 139.04, 143.72, 145.17, 149.34, 153.62, 159.50 (Ar-C and C=N), 163.57 (C=O). MS *m/z* (%): 391 (M^+, 10), 343 (41), 291 (70), 204 (29), 134 (49), 122 (100), 101 (57), 91 (83), 85 (82), 65 (58), 55 (68). Anal. Calcd. for $C_{24}H_{25}NO_4$ (391.46): C, 73.64; H, 6.44; N, 3.58. Found: C, 73.47; H, 6.29; N, 3.36%.

(E)-4-((Furan-2-ylmethylene)amino)phenyl 4-(decyloxy)benzoate (F*10*):

Yield: 84.7%; mp 139-141°C, FTIR (ύ, cm^{-1}): 3089, 2921 (C-H), 1729 (C=O), 1612 (C=N). ^{1}H-NMR (400 MHz, DMSO): *δ*/ppm: 0.79-0.82 (t, 3H, $\underline{CH_3}(CH_2)_7CH_2CH_2O$-), 1.35-1.71 (m, 14H, $CH_3\underline{(CH_2)_7}CH_2CH_2O$-), 1.76-178 (m, 2H, $CH_3(CH_2)_7\underline{CH_2}CH_2O$-), 4.02-4.05 (t, 2H, $CH_3(CH_2)_7CH_2\underline{CH_2}O$-), 6.52-6.54 (t, 1H, furan-H4), 6.67-6.68 (d, 1H, furan-H3), 7.05-7.31 (m, 6H, Ar–H), 7.91-7.92 (d, 1H, furan-H5), 8.01-8.05 (d, 2H, Ar–H), 8.44 (s, 1H, CH=N); ^{13}C-NMR (400 MHz, DMSO): *δ*/ppm: 13.96 (CH_3), 18.14, 22.10, 24.01, 24.94, 25.39, 28.71, 28.97, 34.48, 68.08 (CH_2), 106.68, 114.68, 117.01, 121.94, 122.70, 132.01, 134.01, 139.04,

146.51, 149.01, 149.34, 153.91, 159.50 (Ar-C and C=N), 163.83 (C=O). Anal. Calcd. for $C_{28}H_{33}NO_4$ (447.57): C, 75.14; H, 7.43; N, 3.13. Found: C, 75.05; H, 7.28; N, 3.04%.

(E)-4-((Furan-2-ylmethylene)amino)phenyl 4-(dodecyloxy)benzoate (**F***12*):

Yield: 80.4%; mp125-127 $^{\circ}$C, FTIR (ύ, cm^{-1}): 3077, 2949 (C-H), 1725 (C=O), 1614 (C=N). ^{1}H-NMR (400 MHz, DMSO): δ/ppm: 0.79-0.82 (t, 3H, $\underline{CH_3}(CH_2)_9CH_2CH_2O$-), 1.35-1.71 (m, 16H, $CH_3\underline{(CH_2)_9}CH_2CH_2O$-), 1.76-178 (m, 2H, $CH_3(CH_2)_9\underline{CH_2}CH_2O$-), 4.01-4.06 (t, 2H, $CH_3(CH_2)_9CH_2\underline{CH_2}O$-), 6.53-6.55 (t, 1H, furan-H4), 6.67-6.68 (d, 1H, furan-H3), 7.06-7.36(m, 6H, Ar–H), 7.92 (d, 1H, furan-H5), 8.02-8.04 (d, 2H, Ar–H), 8.44 (s, 1H, CH=N); ^{13}C-NMR (400 MHz, DMSO): δ/ppm: 13.98 (CH_3), 18.03, 22.12, 24.59, 25.41, 28.47, 28.72, 28.97, 29.02, 31.31, 34.31, 67.99 (CH_2), 106.68, 114.68, 117.01, 121.94, 122.70, 132.01, 134.01, 139.04, 146.51, 149.01, 149.34, 153.91, 159.50 (Ar-C and C=N), 163.23 (C=O). MS *m/z* (%): 475 (M^+, 17), 336 (38), 291 (79), 185 (35), 169 (37), 122 (100), 111 (74), 91 (75), 71 (63), 55 (59). Anal. Calcd. for $C_{30}H_{37}NO_4$ (475.62): C, 75.76; H, 7.84; N, 2.94. Found: C, 75.57; H, 7.64; N, 2.83%.

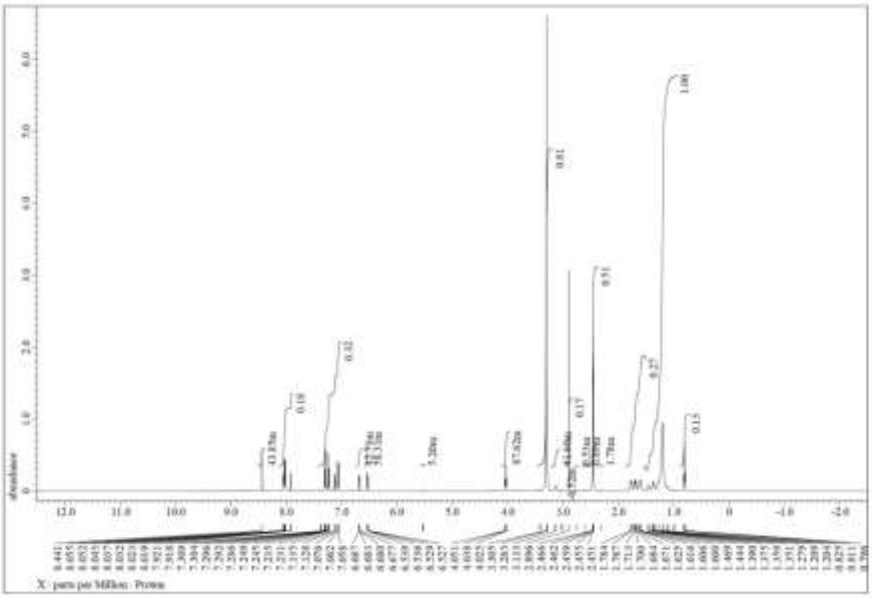

^{1}H-NMR spectra of compound **F10**

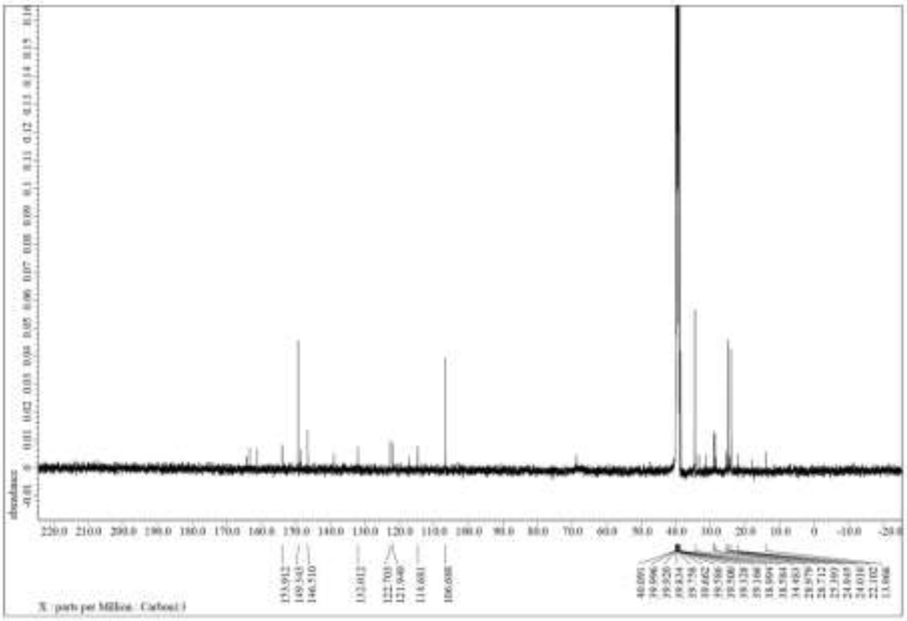

^{13}C-NMR spectra of compound **F10**

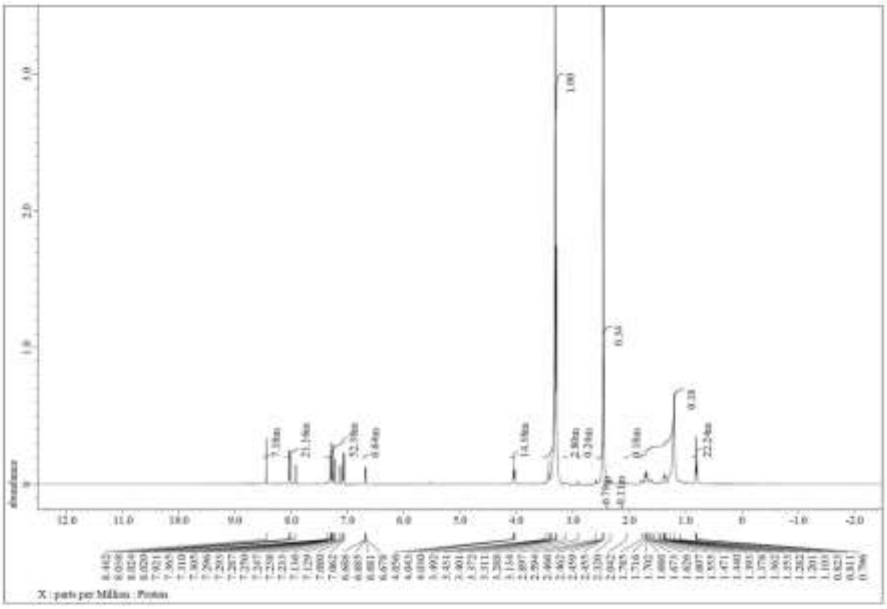

^{1}H-NMR spectra of compound **F12**

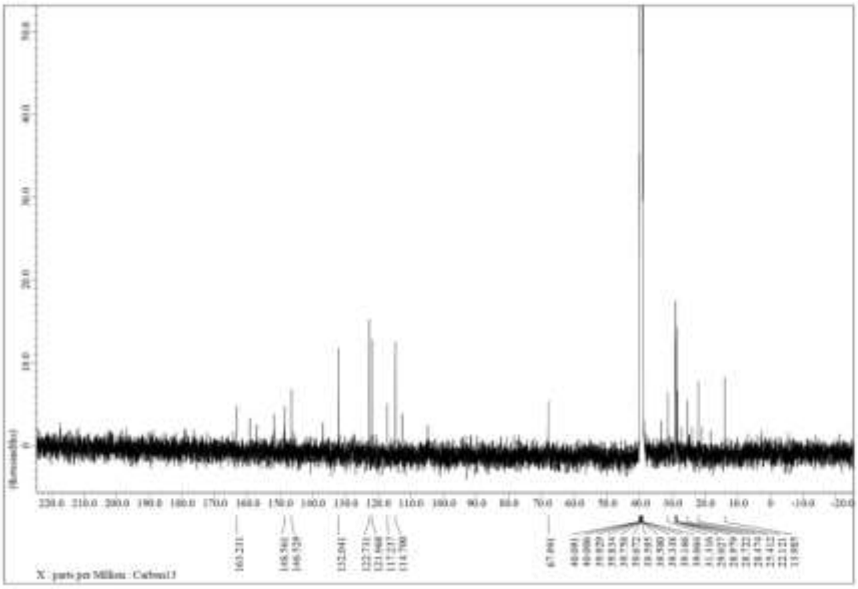

^{13}C-NMR spectra of compound **F12**

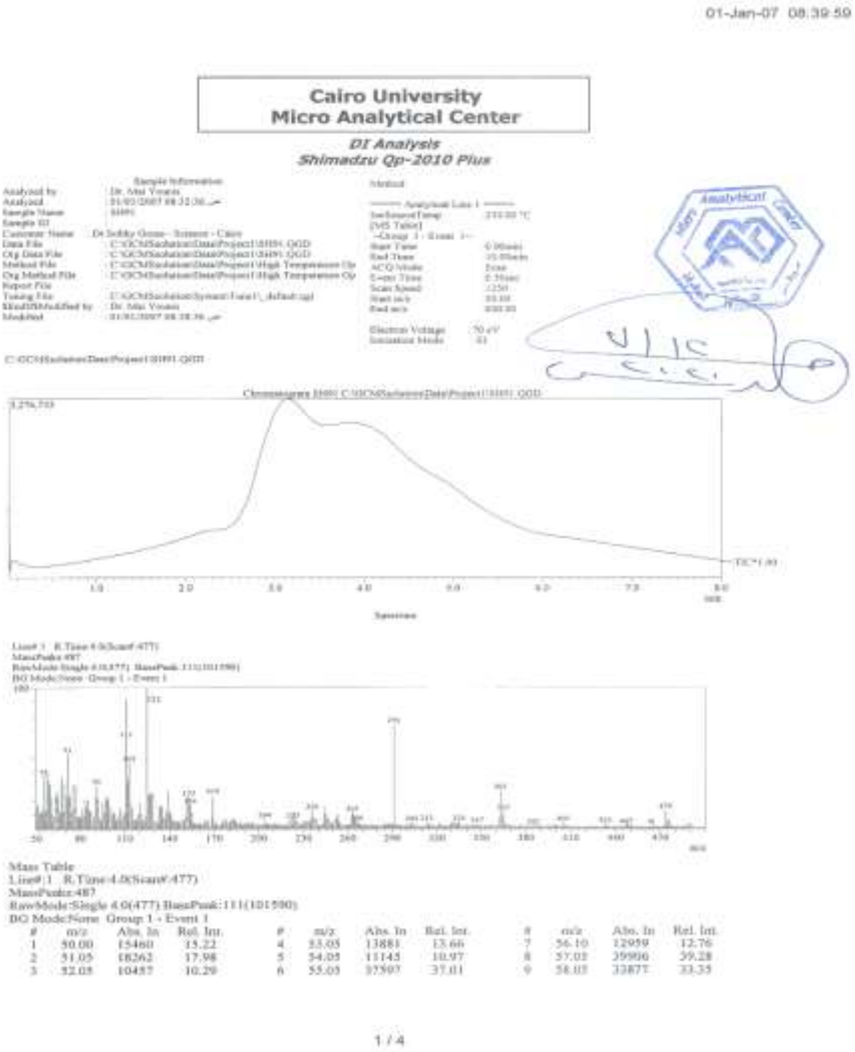

Mass spectra of compound **F12**

4. Characterization

Perkin-Elmer B25 (Perkin-Elmer, Inc., Shelton, CT USA) spectrophotometer was used for infrared spectra measurements. Varian EM 350L 500 MHz spectrometer (Oxford, UK) was used for recording ^{1}HNMR spectra using tetramethyl silane as internal standard in $CDCl_3$; the chemical shift values recorded as δ (in ppm units). Mass spectra were recorded on a GCMS-Q1000-EX Shimadzu and GCMS 5988-A HP spectrometers, the ionizing voltage was 70 eV. Thermo Scientific Flash 2000 CHS/O Elemental Analyzer, Milan, Italy was used for Elemental analyses.

TA Instruments Co. (Q20 Differential Scanning Calorimeter, DSC; USA) was used for recording phase transitions. DSC calibration was carried out using lead and indium to calibrate the melting temperatures and enthalpies. Samples of 2–3 mg were used in aluminum pans for DSC investigation. The heating rate was 10°C/min in nitrogen gas as an inert atmosphere (30 ml/min). All transitions temperatures were measured form the second heating scan.

Transition temperatures for the prepared compounds were checked and phases identified by Polarized optical microscope (POM, Wild, Germany) attached with Mettler FP82HT hot stage.

The mesophase type confirmation of the prepared F12 sample was investigated by means of an X-ray diffraction (XRD) by using Shimadzu Lab X XRD-6000 diffractometer with a Cu-K_α (λ = 1.5406 Å) radiation source in the 2θ range between 20°–80°.

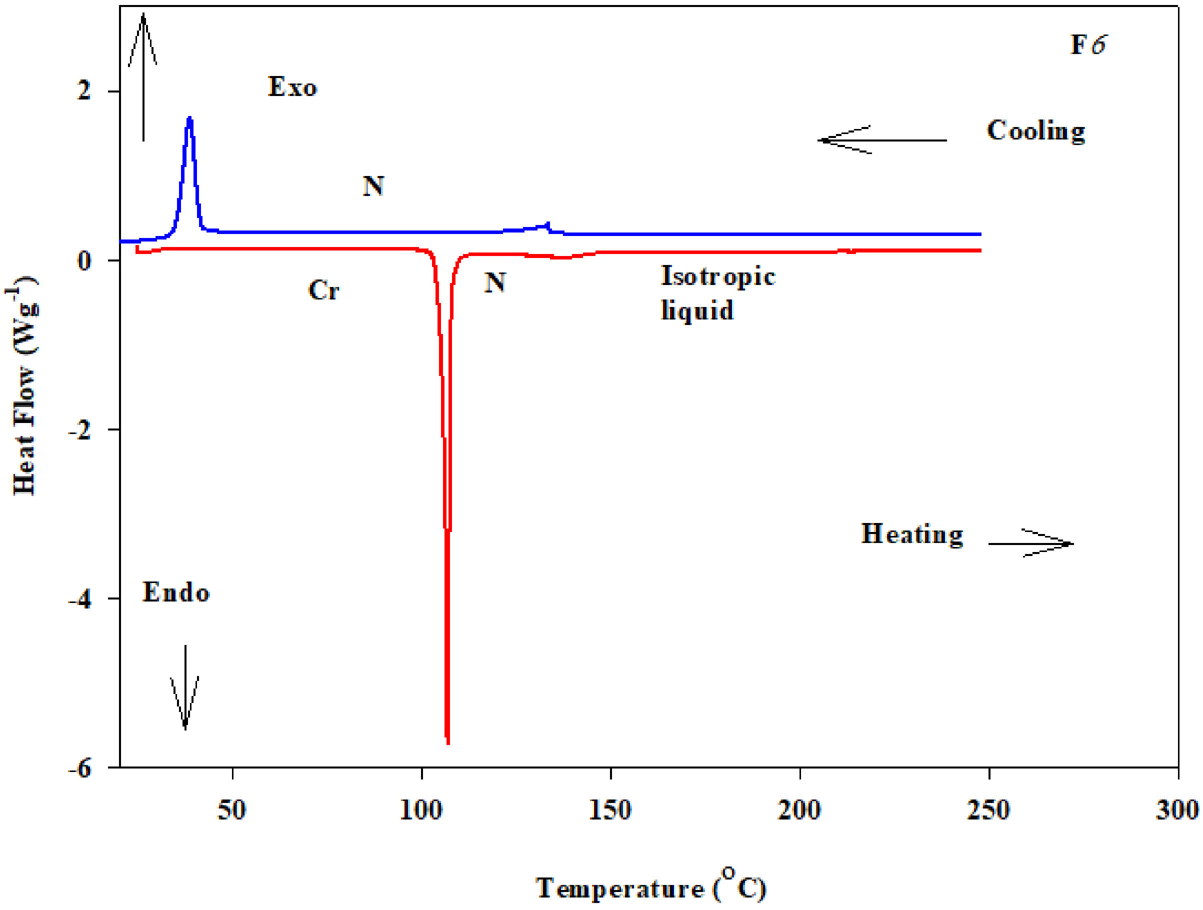

Figure S1: DSC thermograms of **F***6* at a rate of ±10°C/min which are recorded from the second heating and cooling scans**.**

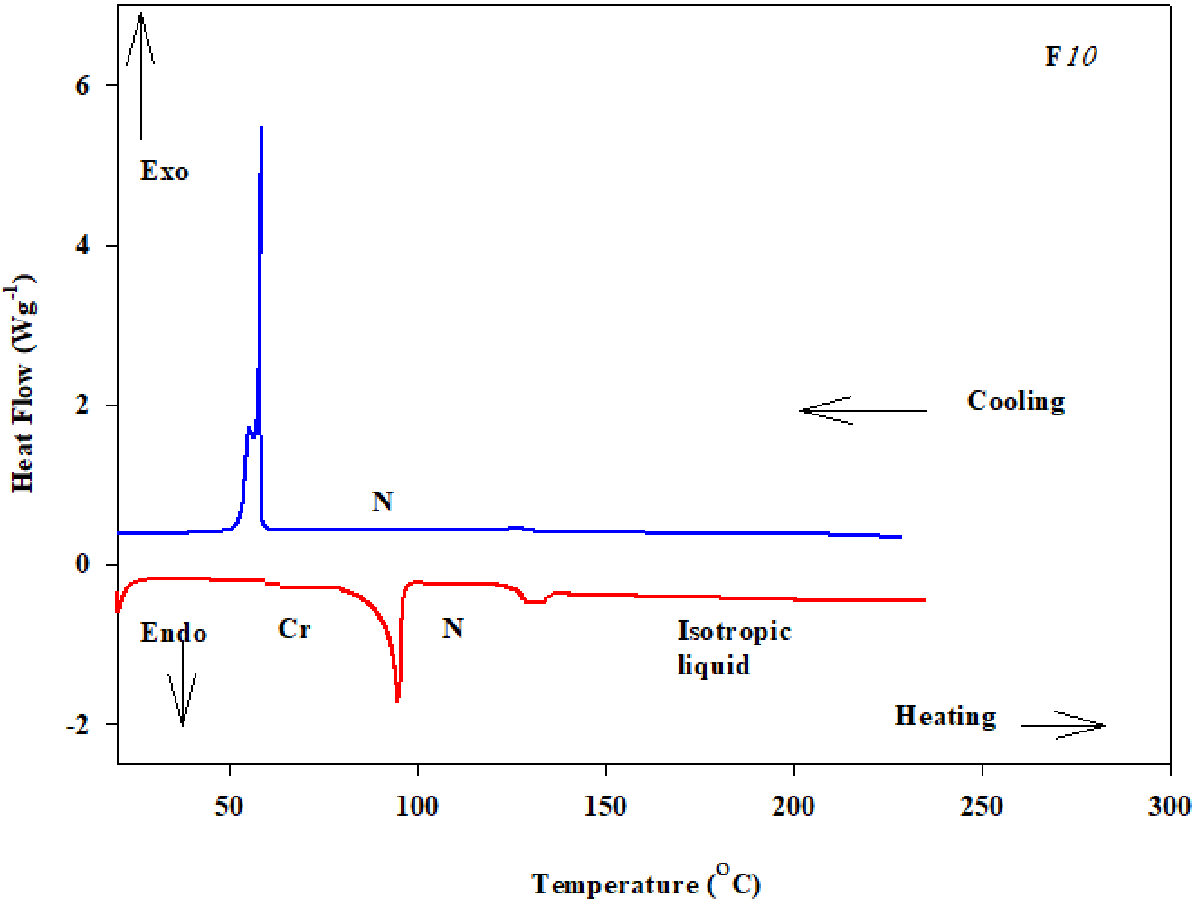

Figure S2: DSC thermograms of **F***10* at a rate of ±10°C/min which are recorded from the second heating and cooling scans**.**

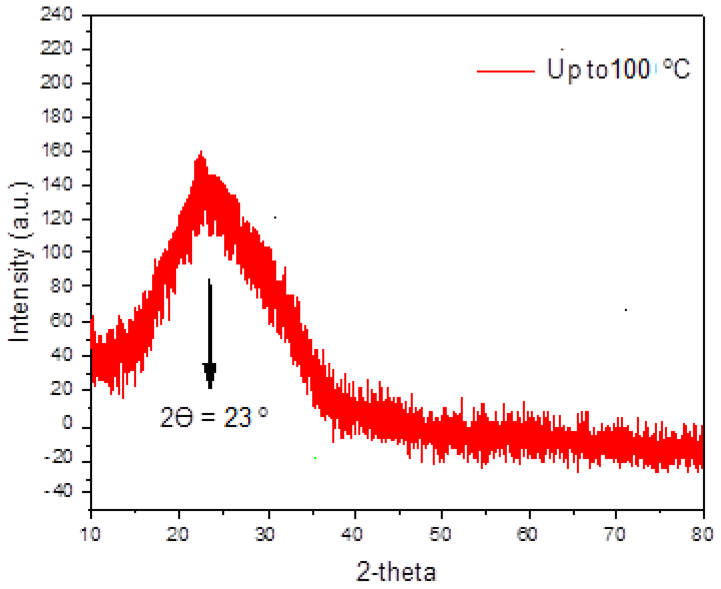

Figure S3. XRD patterns of **F***12* upon cooling from 100 ^{o}C.

References:

i. S. Tighadouni, S. Radi, M. Sirajuddin, M. Akkurt, N. Ö. Matloob Ahmad, Y. N. Mabkhot, T. B. Hadda, In vitro antifungal, anticancer activities and pom analyses of a novel bioactive Schiff base, 4-{[(E)-furan-2-ylmethylidene]amino}phenol: synthesis, characterization and crystal structure. J. Chem. Soc. Pakistan. 38 (2016) 157-165.
